# Supplementary material for: Dual Inhibition of Vacuolar-ATPase and TMPRSS2 Is Required for Complete Blockade of SARS-CoV-2 Entry into Cells
Source: Antimicrob Agents Chemother. 2022 Jun 15;66(7):e00439-22. doi: 10.1128/aac.00439-22 (PMC9295568; doi:10.1128/aac.00439-22)
Supplement: Supplemental file 1 — Fig. S1 to S3 and Table S1. Download aac.00439-22-s0001.pdf, PDF file, 0.8 MB [file aac.00439-22-s0001.pdf]

Supplemental Figure 1

A)

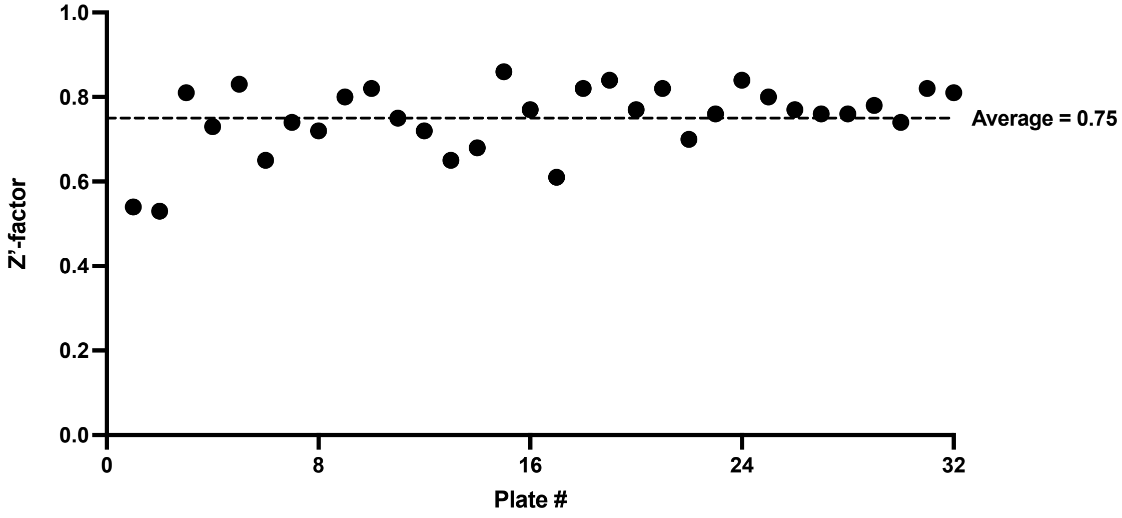

B)

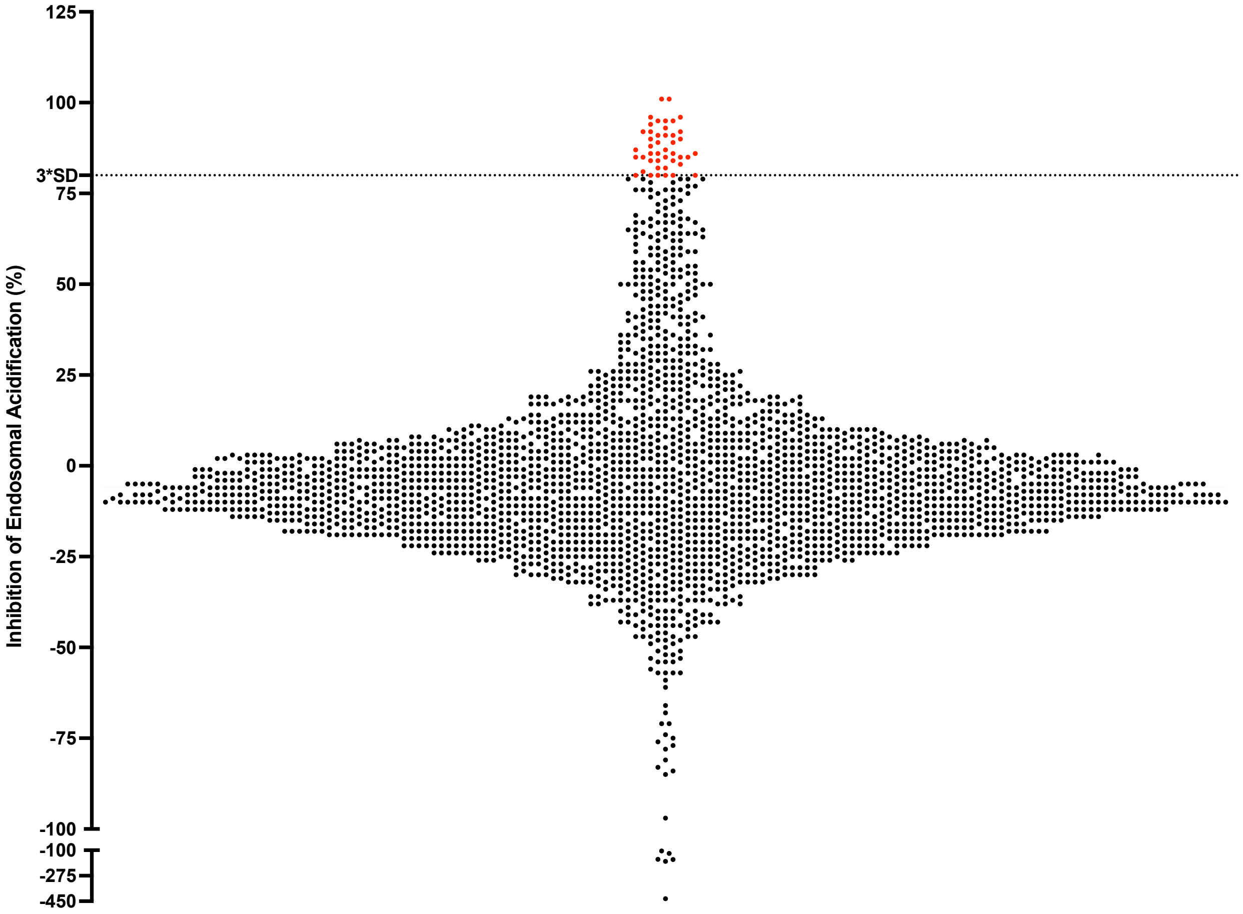

Supplemental Figure 2

A)

Compound Name	Structure
Agaric acid	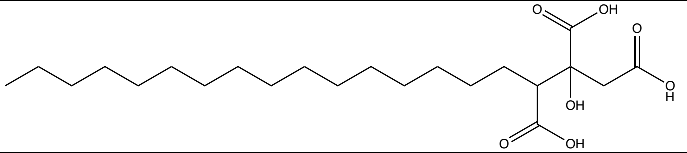
Alexidine	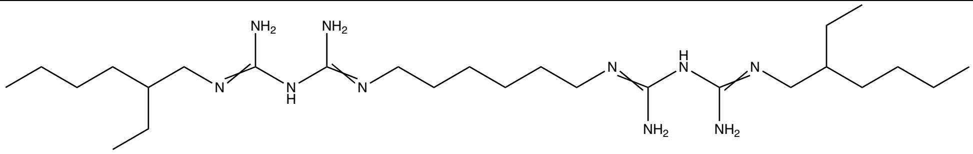
Amsacrine	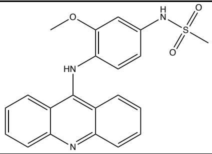
Bazedoxifene	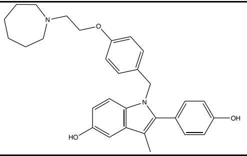
Benzalkonium chloride	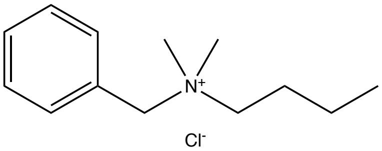
Benzethonium chloride	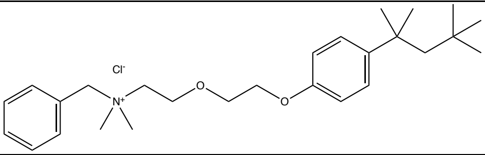
Celastrol	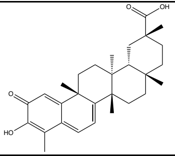
Cetalkonium chloride	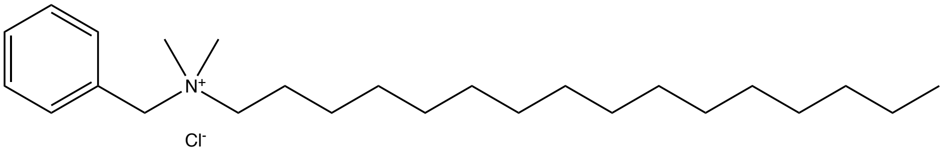
Cinacalcet hydrochloride	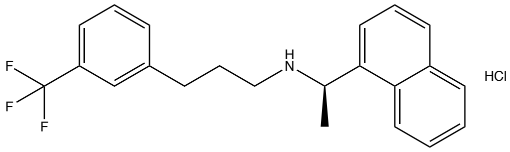
Clofazimine	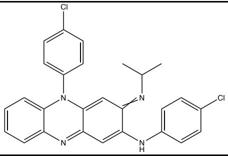

Compound Name	Structure
Clomipramine	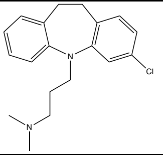
Dapoxetine hydrochloride	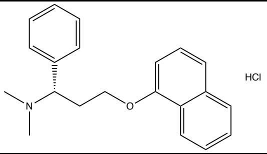
Dronedarone	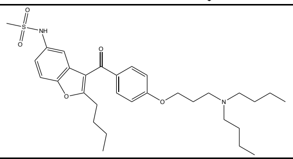
Gambogic acid	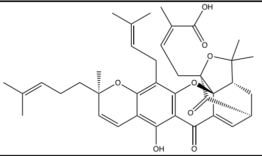
Gossypol	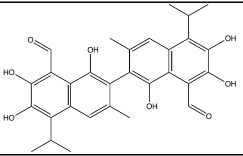
Indapamide	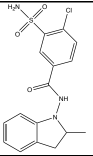
Methylbenzethonium chloride	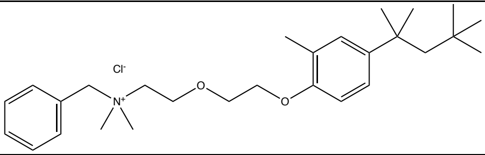
Methylene Blue	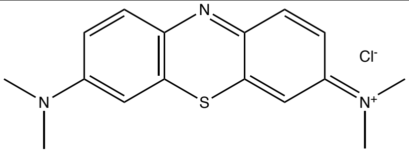
Miconazole	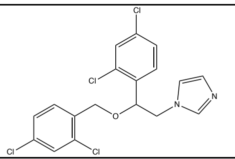
Mitoxantrone	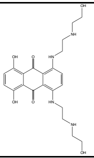

Compound Name	Structure
Perphenazine	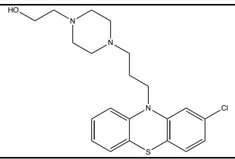
Phenylmercuric acetate	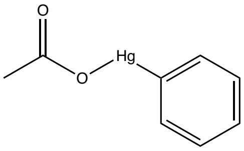
Simvastatin	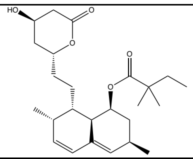
Streptomycin sulfate	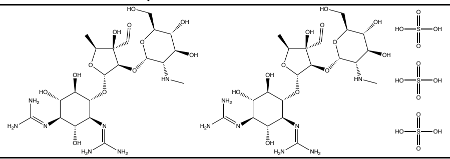
Tamoxifen	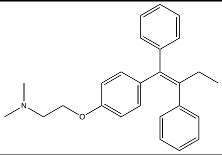
Thimerosal	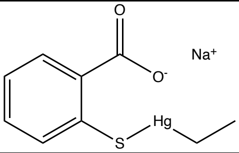
Thioridazine hydrochloride	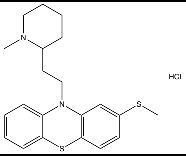
Tioconazole	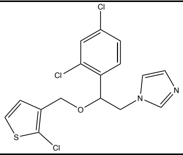
Triclosan	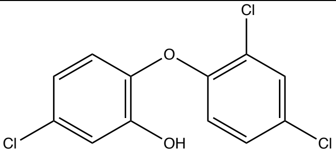

B)

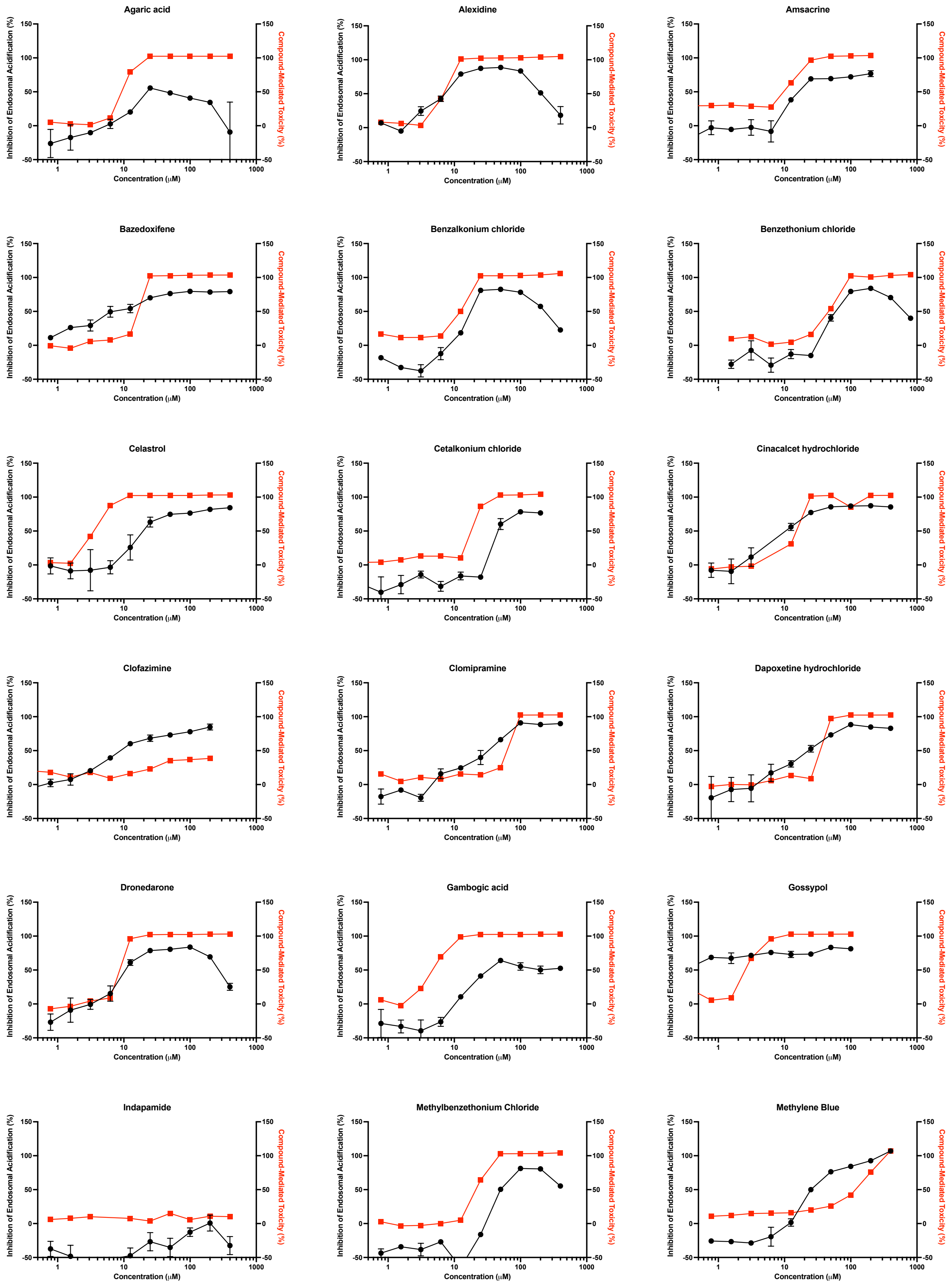

Agaric acid
Alexidine
Amsacrine
Bazedoxifene
Benzalkonium chloride
Benzethonium chloride
Celastrol
Cetalkonium chloride
Cinacalcet hydrochloride
Clofazimine
Clomipramine
Dapoxetine hydrochloride
Dronedarone
Gambogic acid
Gossypol
Indapamide
Methylbenzethonium Chloride
Methylene Blue
Inhibition of Endosomal Acidification (%)
Compound-Mediated Toxicity (%)
Concentration (μM)

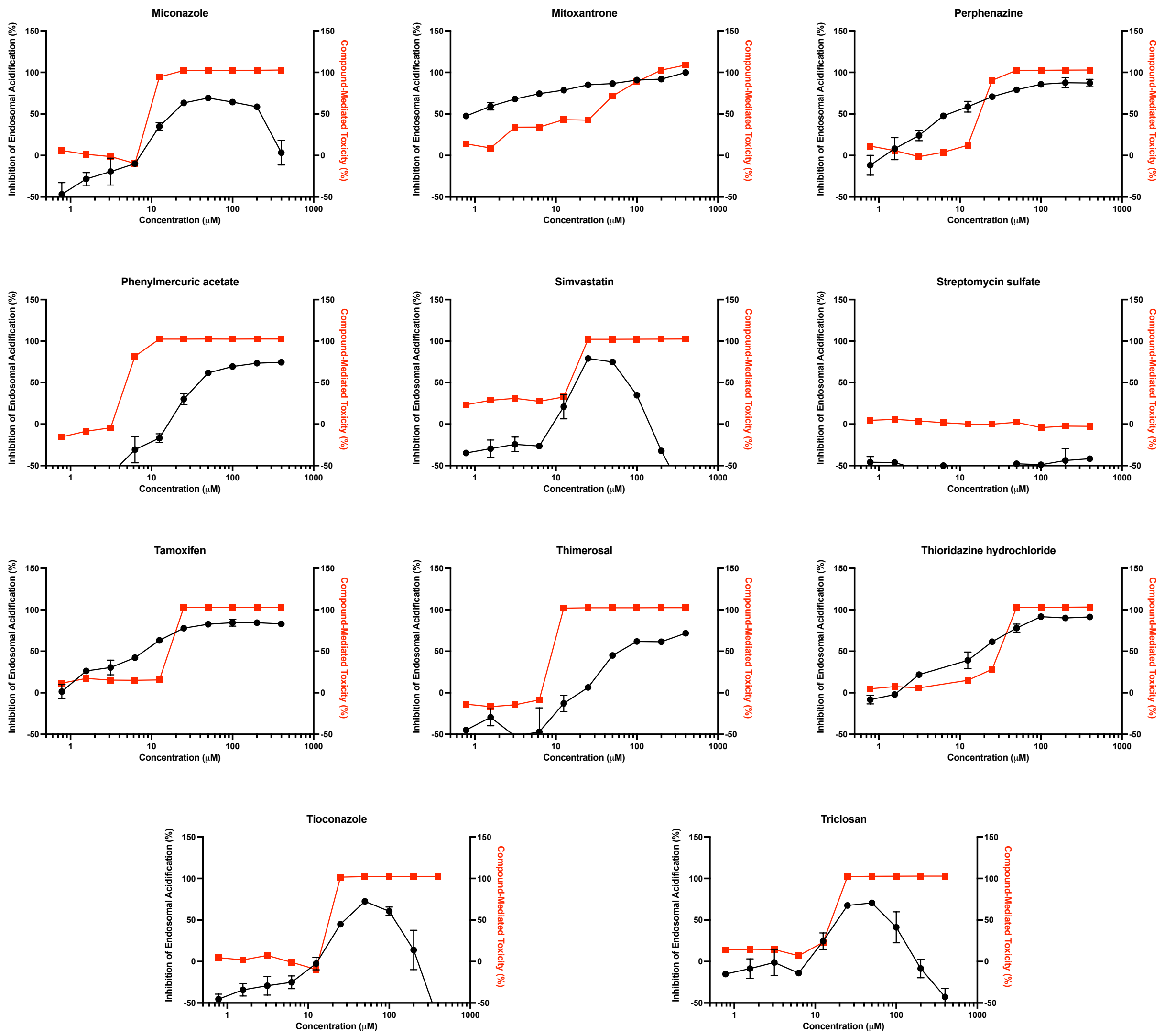
Miconazole
Mitoxantrone
Perphenazine
Phenylmercuric acetate
Simvastatin
Streptomycin sulfate
Tamoxifen
Thimerosal
Thioridazine hydrochloride
Tioconazole
Triclosan
Inhibition of Endosomal Acidification (%)
Compound-Mediated Toxicity (%)
Concentration (μM)
150
100
50
0
-50
1
10
100
1000

Supplemental Figure 3

A) VERO Cells

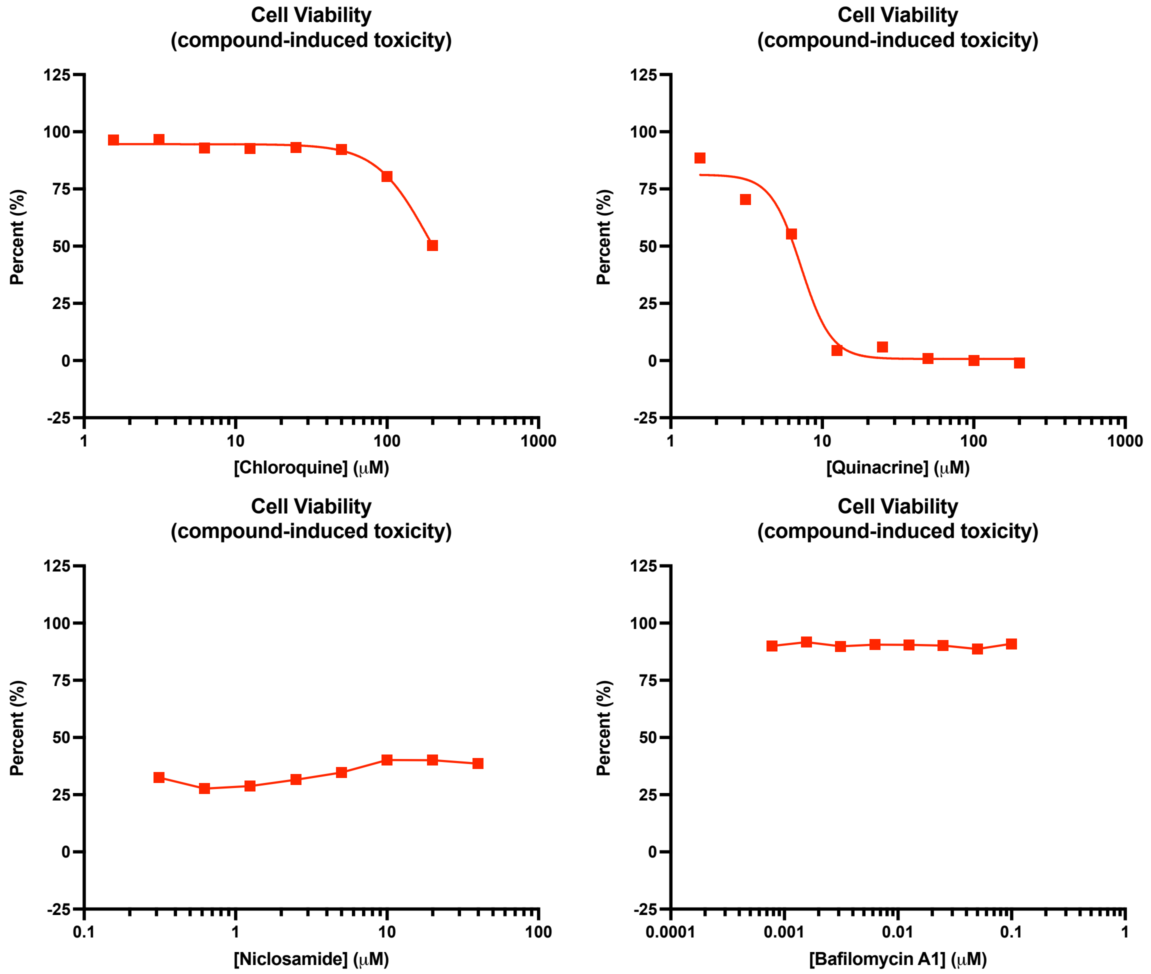

B) Calu-3 Cells

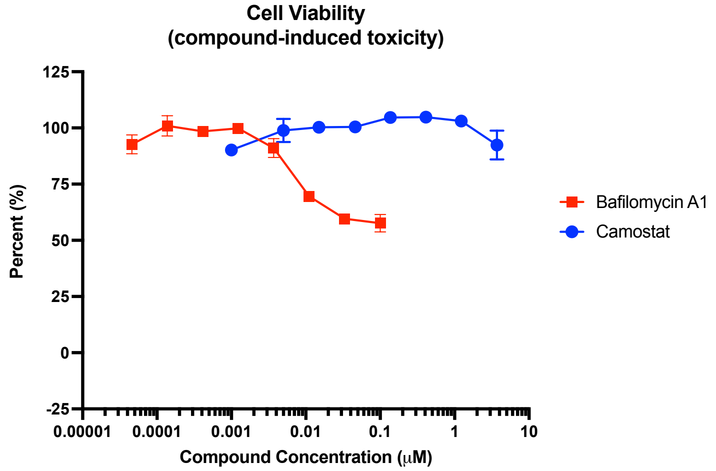

Supplemental Table 1: Compound screening data

Molecule Name	Percent Inhibition (%)
STREPTOMYCIN SULFATE	101
INDAPAMIDE	101
DIHYDROCELASTROL	96
TRICLOSAN	96
GENTIAN VIOLET	95
ALEXIDINE HYDROCHLORIDE	95
SULOCTIDIL	94
TIOCONAZOLE	94
PERPHENAZINE	93
CELASTROL	92
THIORIDAZINE HYDROCHLORIDE	92
PERHEXILINE MALEATE	92
CINACALCET HYDROCHLORIDE	91
NONOXYNOL-9	91
DAPOXETINE HYDROCHLORIDE	91
GAMBOGIC ACID	90
CETALKONIUM CHLORIDE	90
AGARIC ACID	89
BAZEDOXIFENE ACETATE	89
BENZALKONIUM CHLORIDE	88
AMSACRINE	87
PHENYLMERCURIC ACETATE	86
DICHLOROPHEN	86
TRIFLUPROMAZINE HYDROCHLORIDE	86
LYNESTRENOL	86
alpha-MANGOSTIN	86
METHYLENE BLUE	85
METHYLBENZETHONIUM CHLORIDE	85
DRONEDARONE HYDROCHLORIDE	85
DALBERGIONE	85
ISOPOMIFERIN	85
TOLONIUM CHLORIDE	84
HEXETIDINE	84
SIMVASTATIN	84
CLOMIPRAMINE HYDROCHLORIDE	83
TAMOXIFEN CITRATE	82
THIMEROSAL	81
MICONAZOLE NITRATE	81
THIOTHIXENE	80
GOSSYPOL	80
MITOXANTRONE HYDROCHLORIDE	80

CLOFAZIMINE	80
CETYLPYRIDINIUM CHLORIDE	80
BENZETHONIUM CHLORIDE	80
CETRIMONIUM BROMIDE	79
ISOCONAZOLE NITRATE	79
DIGITONIN	79
4-NONYLPHENOL	79
PIPERACETAZINE	78
FLUPHENAZINE HYDROCHLORIDE	78
TRIMEPRAZINE TARTRATE	78
TRIFLUOPERAZINE HYDROCHLORIDE	77
CHLOROPHYLLIDE Cu COMPLEX Na SALT	77
AMLODIPINE BESYLATE	76
PRAZOSIN HYDROCHLORIDE	76
ROBENIDINE HYDROCHLORIDE	76
SULCONAZOLE NITRATE	76
FARNESOL	76
ECONAZOLE NITRATE	76
NICLOSAMIDE	75
PROMAZINE HYDROCHLORIDE	75
TOTAROL	74
SERTRALINE HYDROCHLORIDE	74
FENDILINE HYDROCHLORIDE	73
PROCHLORPERAZINE EDISYLATE	73
TOMATINE	72
BEPRIDIL HYDROCHLORIDE	71
PYRONARIDINE TETRAPHOSPHATE	70
ETHOPROPAZINE HYDROCHLORIDE	70
DESLORATIDINE	70
HYCANTHONE	69
AMINACRINE	69
CLOMIPHENE CITRATE	69
PRIMAQUINE PHOSPHATE	68
RAFOXANIDE	68
TOREMIPHENE CITRATE	68
FLUOXETINE	67
18-AMINOABIETA-8,11,13-TRIENE SULFATE	67
CYPROHEPTADINE HYDROCHLORIDE	67
EBASTINE	67
BITHIONATE SODIUM	67
ACEPROMAZINE MALEATE	67
NEROLIDOL	66
TOTAROL-19-CARBOXYLIC ACID, METHYL ESTER	66
PAROXETINE HYDROCHLORIDE	65

CHLORPROMAZINE	65
CANTHARIDIN	65
BEXAROTENE	64
GUAIAZULENE	64
TERCONAZOLE	64
ARACHIDONIC ACID	64
BENAZEPRIL HYDROCHLORIDE	64
TESTOSTERONE PROPIONATE	64
MEFLOQUINE	64
METHYL DEOXYCHOLATE	63
IDEBENONE	63
VORTIOXETINE HYDROBROMIDE	63
QUINACRINE HYDROCHLORIDE	63
APREPITANT	62
CARVEDILOL PHOSPHATE	62
ACETOPHENAZINE MALEATE	62
PENFLURIDOL	61
MAPROTILINE HYDROCHLORIDE	60
AMPHOTERICIN B	60
DULOXETINE HYDROCHLORIDE	59
ABAMECTIN (avermectin B1a shown)	59
TILORONE	59
PROADIFEN HYDROCHLORIDE	59
AZELASTINE HYDROCHLORIDE	59
DROFENINE HYDROCHLORIDE	58
PYRITHIONE ZINC	58
BENZYDAMINE HYDROCHLORIDE	58
METITEPINE MALEATE	58
CHLORPROTHIXENE HYDROCHLORIDE	57
PROMETHAZINE HYDROCHLORIDE	56
CLOSANTEL	56
RALOXIFENE HYDROCHLORIDE	56
TRIMIPRAMINE MALEATE	56
CARVEDILOL	55
OXYCLOZANIDE	55
PROFLAVINE HEMISULFATE	54
CHICAGO SKY BLUE	54
PIMOZIDE	54
LOPERAMIDE HYDROCHLORIDE	54
IMIPRAMINE HYDROCHLORIDE	54
CARAZOLOL	54
DIPERODON HYDROCHLORIDE	54
METERGOLINE	53
AMITRIPTYLINE HYDROCHLORIDE	53

MEBHYDROLIN NAPHTHALENESULFONATE	52
AMIODARONE HYDROCHLORIDE	52
CYCLANDELATE	52
NEBIVOLOL HYDROCHLORIDE	52
MEPARTRICIN	52
ACRIFLAVINIUM HYDROCHLORIDE	51
HALOPERIDOL	51
FLUPIRTINE MALEATE	50
DESIPRAMINE HYDROCHLORIDE	50
BENZTROPINE MESYLATE	50
ASTEMIZOLE	50
PIZOTYLINE MALATE	50
ACRISORCIN	50
BIFONAZOLE	50
TETRANDRINE	50
BROMPERIDOL	50
TERFENADINE	50
ANDROSTERONE ACETATE	50

SUPPLEMENTAL FIGURE AND TABLE LEGENDS

Supplemental Figure 1. High throughput screen of the Spectrum Collection library to identify inhibitors of endosomal acidification. (A) The calculated Z-factor from each of the 32 plates from the screen. (B) High throughput inhibition of endosomal acidification screen data. each circle represents the inhibition of a unique compound from the Spectrum Collection library tested at 40 μM. The dashed line represents the calculated hit cut off (mean plus three times the standard deviation of the data), with the red circles demonstrating inhibition values greater than the cut off. All experiment were conducted in singlet (n=1).

Supplemental Figure 2. Dose response inhibition of endosomal acidification and compound-mediated toxicity of compounds above the hit cut off. (A) Chemical structure of the compounds selected from the high throughput screen for further testing. (B) Comparison of the inhibition of endosomal acidification and compound-mediated toxicity by each tested compound across a dose titration range. Inhibition of endosomal acidification experiments were conducted in duplicate (n=2) and cell toxicity experiments were conducted in singlet (n=1).

Supplemental Figure 3. Compound-mediated toxicity of endosomal acidification inhibitors. (A) Compound-mediated toxicity against Vero cells by endosomal acidification inhibitors. (B) Compound-mediated toxicity against Calu-3 cells.

Supplemental Table 1. Inhibition of endosomal acidification by each compound from the Spectrum Collection library.
